# Sex-specific differences in disease severity and outcomes in left ventricular heart failure: a nationwide cohort study

**DOI:** 10.1007/s00392-026-02876-9

**Published:** 2026-02-23

**Authors:** Anastasia Janina Hobbach, Jannik Feld, Jeanette Köppe, Jürgen Reinhard Sindermann, Holger Reinecke

**Affiliations:** 1 Department of Cardiology I, Peripheral Vascular Disease and Heart Failure University Hospital, Albert-Schweizer Campus 1, 48149 Münster, Germany; 2 Institute of Biostatistics and Clinical Research, University of Münster, Münster, Germany

**Keywords:** Left ventricular heart failure, Sex differences, NYHA classification, In-hospital mortality, Germany

## Abstract

**Aims:**

Left ventricular heart failure (LVHF) remains a major contributor to morbidity and mortality, with sex-specific differences. This study investigates the influence of sex and New York Heart Association (NYHA) classification on clinical outcomes and healthcare costs.

**Methods and results:**

We analyzed data from 2,616,462 LVHF hospitalization cases in Germany (2014–2022), sourced from the Research Data Centre of the Federal Statistical Office and Statistical Offices of the Federal States (DESTATIS). Cases were stratified by sex and NYHA stages. Baseline characteristics, comorbidities, in-hospital outcomes, and healthcare costs were assessed. Multivariable logistic regression evaluated in-hospital survival. Women were more frequently diagnosed at earlier NYHA stages; men dominated in advanced stages. In-hospital mortality was higher among women (8.34% (108,461) vs. 7.90% (103,883)). However, stratified analyses showed higher mortality rates for men across most age and NYHA groups, except for men aged 80–89 in NYHA I, < 40 in NYHA III, and 40–59 in NYHA IV. Despite being younger and hospitalized for shorter or equal durations, men incurred higher costs in NYHA III (2,931.34 vs. 2,922.67) and NYHA IV (2,960.72 vs. 2,923.36). NYHA stage was the strongest predictor of in-hospital mortality (NYHA II: OR 1.596; NYHA III: OR 5.290; NYHA IV: OR 22.533]; all *p* < 0.001), while female sex (OR 0.841) and obesity (OR 0.751) were associated with lower mortality.

**Conclusion:**

LVHF outcomes and costs differ by sex and NYHA stage. Though women had higher overall mortality, men showed worse outcomes in most subgroups. These findings stress the importance of sex- and stage-specific LVHF management strategies.

**Graphical Abstract:**

This nationwide study analyzed over 2.6 million hospital admissions for LVHF in Germany between 2014 and 2022. Female cases were more often diseased with CKD, AHT and AFl and had higher overall-mortality rates, while male cases had higher healthcare costs and poorer survival in most age- and NYHA stratified groups. These findings underscore the importance of sex- and severity-specific LVHF management strategies.

Atrial flutter/fibrillation (AFl), arterial hypertension (AHT), left ventricular heart failure (LVHF), chronic kidney disease (CKD), New York Heart Association (NYHA).

**Figure:**
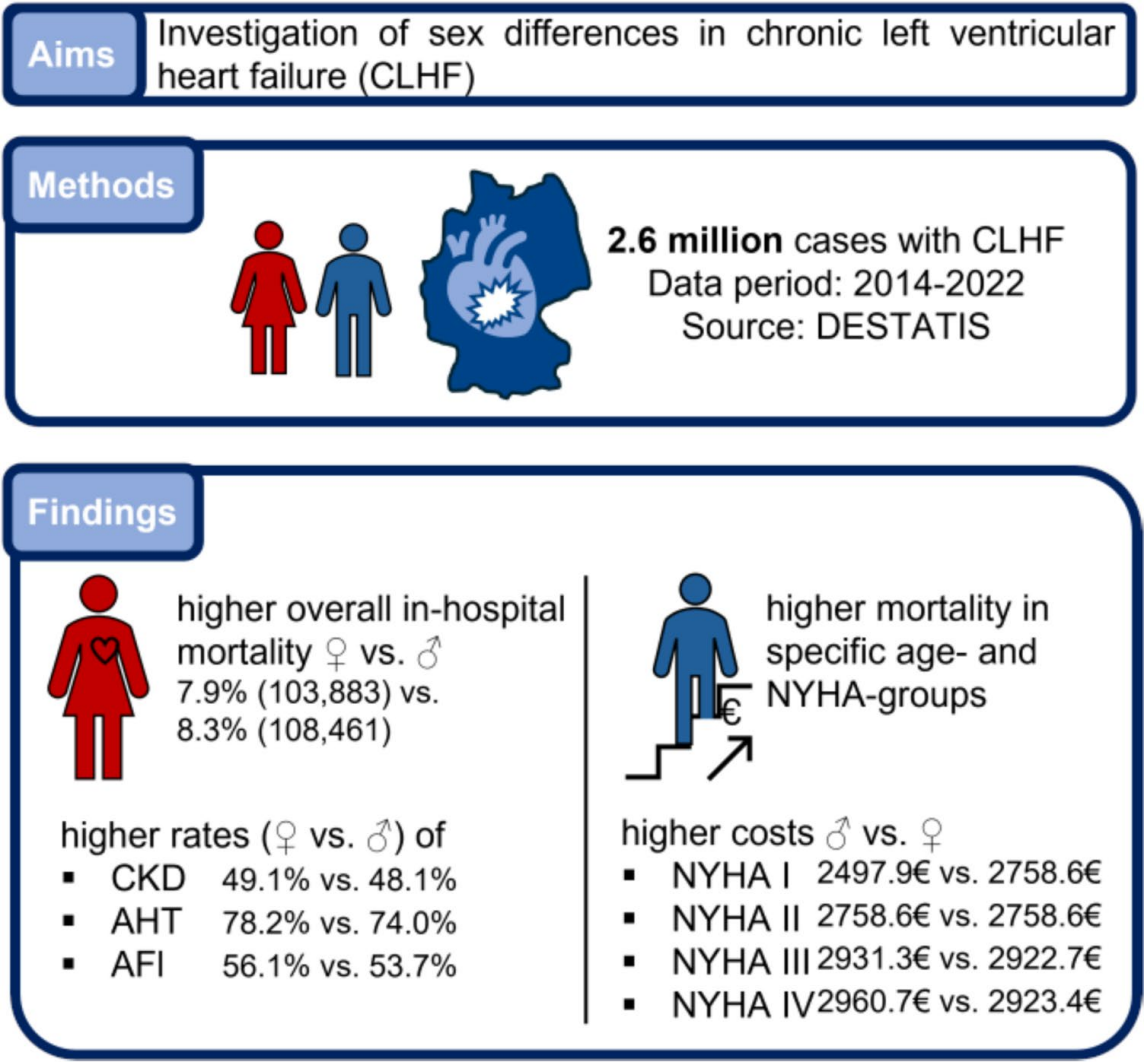

**Supplementary Information:**

The online version contains supplementary material available at 10.1007/s00392-026-02876-9.

## Introduction

Left ventricular heart failure (LVHF) remains a leading cause of morbidity and mortality worldwide, imposing a substantial burden on healthcare systems. The classification of LVHF according to the New York Heart Association (NYHA) provides a widely accepted framework for assessing disease severity based on symptomatology and functional limitations. According to the 2023 ESC Guidelines for the diagnosis and treatment of heart failure (HF), the diagnosis of HF is based on the presence of typical symptoms (e.g., breathlessness, fatigue, ankle swelling) and signs (e.g., elevated jugular venous pressure, pulmonary crackles, peripheral edema) caused by a structural and/or functional cardiac abnormality, corroborated by objective evidence of cardiac dysfunction and/or elevated natriuretic peptides. HF is further classified according to left ventricular ejection fraction (LVEF) into HF with reduced LVEF (HFrEF; LVEF ≤ 40%), HF with mildly reduced LVEF (HFmrEF; LVEF 41–49%), and HF with preserved LVEF (HFpEF; LVEF ≥ 50%), each of which has distinct epidemiological, pathophysiological, and prognostic profiles [1]. While this classification is integral to LVHF management, its prognostic implications may differ between patient subgroups, particularly when considering sex-specific disparities.

Sex profoundly influences the epidemiology, pathophysiology, and clinical management of LVHF. These distinctions are driven by a complex interplay of hormonal variations, comorbidities, and divergent responses to therapies [2]. However, beyond biological determinants, sex disparities extend to the diagnosis, treatment, and prognosis of LVHF. Evidence suggests that women with cardiovascular diseases are frequently underdiagnosed and undertreated [2–6]. In particular, women with LVHF receive guideline-recommended therapies less frequently and are significantly less likely to undergo heart transplantation [7].

Despite growing recognition of these sex-related disparities, their interaction with LVHF severity, as classified by NYHA, remains insufficiently explored on a population level. This study aims to systematically examine the impact of sex and NYHA class on key in-hospital outcomes in LVHF, including mortality and length of hospital stay. Using comprehensive nationwide data from the Research Data Centre of the Federal Statistical Office and Statistical Offices of the Federal States (DESTATIS), we analyzed whether sex-specific differences in disease progression manifest across different NYHA classes and identified potential contributing factors. By providing large-scale, real-world evidence, our findings may inform future strategies to optimize LVHF management and reduce inequities in care.

## Methods

### Data source and study population

In Germany, all hospitals are legally required to report data on inpatient hospitalizations to the Institute for the Hospital Remuneration System (Institut für das Entgeltsystem im Krankenhaus, InEK; Siegburg, Germany; http://www.g-drg.de) since 2002. These data have been largely made available for research purposes by DESTATIS (Statistisches Bundesamt, DESTATIS; https://www.destatis.de; RDC of the Federal Statistical Office and Statistical Offices of the Federal States, [10.21242/23141.2014.00.00.6.1.0 to 10.21242/23141.2022.00.00.6.1.0], own calculations). For this study, we used hospital data from DESTATIS, covering the period from 2014 to 2022 (graphical abstract). The investigation conforms with the principles outlined in the *Declaration of Helsinki* (Br Med J 1964; ii:177). Patient consent was waived due to the retrospective character with anonymized data of this study.

The dataset comprises all inpatient cases treated in German hospitals, except for admissions to psychiatric or psychosomatic units. All analyses were conducted by DESTATIS using our pre-approved scripts, as direct access to the anonymized dataset was not permitted. Script development and validation were carried out using a synthetic dataset reflecting the structure of the original data. As hospitals in Germany are legally required to submit standardized administrative data on all inpatient cases to the DESTATIS, the dataset covers virtually all hospitalizations nationwide. Further methodological details can be found in previous publications [8–10].

The dataset included records of 2,616,462 hospitalization cases of patients with the primary diagnosis of LVHF (ICD-10-GM I50.11-I50.14) between 1 January 2014, and 31 December 2022. In line with the ICD-10-GM coding system, these codes specify the NYHA functional class of LVHF but do not differentiate between acute and chronic forms. Only inpatient cases with LVHF as the primary diagnosis were included; cases with LVHF as a secondary diagnosis were not part of the analysis, as our aim was to study patients admitted primarily for LVHF. All comorbidities and procedures were identified using the International Classification of Diseases, 10th Revision, German Modification (ICD-10-GM) (Online Resource 1) and the Operationen- und Prozedurenschlüssel (OPS) codes (Online Resource 2). If a relevant code was not documented within the hospital stay, the variable was set to zero. Cases were stratified by LVHF severity based on the NYHA classification and further categorized by sex.

The number of cases was stratified by NYHA and sex (Online Resource 3). Baseline characteristics were extracted (Online Resource 3), including cardiovascular risk factors (CVRF), such as arterial hypertension (AHT), diabetes mellitus (DM), dyslipidemia, obesity, smoking. Additionally, cardiovascular comorbidities were assessed, including coronary heart disease (CHD), peripheral artery disease (PAD), chronic kidney disease (CKD), atrial flutter/fibrillation (AFl), ischemic stroke, hemorrhagic stroke, secondary diagnosis of chronic left/right ventricular heart failure (cL/RVHF), previous stroke, previous coronary artery bypass grafting (CABG), cerebrovascular disease (CeVD), previous valve implantation, previous myocardial infarction (MI), previous percutaneous coronary intervention (PCI)). Recorded interventions associated with comorbidities comprised left-heart catheterization (LCH), PCI, drug-eluting stent (DES) implantation, use of a drug eluting balloon (DEB), plain old balloon angioplasty (POBA), and CABG. Complications included cardiogenic shock, acute kidney injury (AKI), renal replacement therapy, the combination of resuscitation and left ventricular (LV) support, implantation of an assist device (e.g. extracorporeal membrane oxygenation (ECMO), IMPELLA, intra-aortic balloon pump (IABP)) and mechanical ventilation.

Outcomes of interest encompassed the implantation of uni- or biventricular intracorporeal pumps, total artificial heart implantation (total heart replacement), orthotopic or heterotopic (assist heart) heart transplantation, heart-lung transplantation and in-hospital mortality.

The median healthcare costs, length of hospitalization, and age at hospitalization were analyzed separately by NYHA stage and sex (Online Resource 4). For estimation of costs, the total financial reimbursements per case that the hospitals received was used. These payments are made by health insurance providers based on the services delivered.

A detailed temporal trend analysis of in-hospital outcomes in cases with LVHF over the study period (2014–2022) using the same DESTATIS dataset has been published separately [10] and is therefore not repeated in the present work.

### Statistics

This study included all inpatient cases of LVHF in Germany from 2014 to 2022. Comparisons of categorical variables between groups (e.g., sex or NYHA stages) were conducted using a two-sided Chi-square test, whereas continuous variables were analyzed using the two-sided Wilcoxon test.

All statistical analyses were exploratory in nature, and p-values were interpreted accordingly. Specifically, all p-values were two-sided, with values below 0.05 considered indicative of a statistically relevant difference. Model performance was assessed by calculating the concordance index (c-statistic) and Somers’ D, and by performing the Hosmer–Lemeshow goodness-of-fit test. The c-statistic, equivalent to the area under the receiver operating characteristic curve (AUC), reflects the model’s discriminatory ability, whereas the Hosmer–Lemeshow test evaluates calibration. Statistical analyses were performed using SAS software (SAS 9.3, SAS Institute Inc., Cary, NC, USA) and R version 4.3.2 (2023–10–31).

## Results

### Study population

Our study included a total of 2,616,462 hospital admissions due to the primary diagnosis of LVHF, with an overall balanced sex distribution (male: 50.29% (1,315,700) vs. female: 49.71% (1,300,762); Online Resource 3). In the same observation period, there were 1,394,668 cases coded with a primary diagnosis of RVHF (ICD-10-GM I50.0, I50.1) and 26,247 cases with unspecified HF (I50.9). 1,359,635 of these represent global or secondary RVHF due to LVHF (ICD-10-GM I50.01). These cases were not further analyzed in the present study, as our predefined research question specifically focused on primary LVHF.

The dataset predominantly comprised individuals with advanced LVHF, as more than half of the cases (52.21%) were classified as NYHA stage IV, whereas fewer than 1% (0.98%) were asymptomatic (NYHA I). The proportion of female cases was higher in NYHA stages I and II (NYHA I: 55.95% female, *p* < 0.0001; NYHA II: 51.16% female, *p* < 0.000; Online Resource 3), whereas male cases were more frequently represented in NYHA stage III (51.14% male, *p* < 0.0001; Online Resource 3). In end-stage LVHF (NYHA IV), sex distribution was nearly equal (49.92% vs. 50.08%, *p* = 0.056; Online Resource 3).

### Baseline characteristics

The prevalence of CVRF in the cohort was substantial (Online Resource S3). Specifically, AHT was present in 76.11% of cases, DM in 38.19%, and dyslipidemia in 30.18%. Cardiovascular comorbidities were also highly prevalent, with CKD (48.60%) and CHD (32.70%) being the most common.

With the exception of mortality, recorded outcomes were observed in only a small proportion of cases (Online Resource 3). Notably, < 0.0001% of cases received a biventricular intracorporeal pump, and only 2,453 (0.09%) underwent implantation of a univentricular intracorporeal pump. Heart transplantation was performed in 0.01% of cases. Among the included cases, 67,420 (2.6%) had a concomitant diagnosis of acute MI (ICD-10-GM I21.x or I22.x) during the same hospitalization for LVHF. Overall, the in-hospital mortality rate was 8.12%, with a mean age at death of 82.85 ± 8.88 years. Male hospitalization cases were younger on average (80.78 ± 9.25 years) than women (84.83 ± 8.02 years).

### Sex-specific differences

In the overall cohort, without stratification by NYHA class, most CVRF, comorbidities, and clinical events occurred more often in male cases compared to female cases (Tables 1, 2). However, notable exceptions included CKD (♂: 48.10% vs. ♀: 49.10%), obesity (♂: 10.85% vs. ♀: 11.21%), AFl (♂: 53.67% vs. ♀: 56.08%), AHT (♂: 74.07% vs. ♀: 78.21%), and prior stroke (♂: 2.78% vs. ♀: 2.91%), where female cases were more frequently affected (Table 1). Importantly, in-hospital mortality was also higher among female cases (♂: 7.9% vs. ♀: 8.34%) (Table 2).

**Table 1:** Cardiovascular risk factors, cardiovascular comorbidities, recorded interventions, and complications. Prevalence of cardiovascular risk factors, cardiovascular comorbidities, recorded interventions, complications, and outcomes relative to the total number of male and female cases in hospital admissions for LVHF, presented in absolute and relative numbers, stratified by sex. *AKI* Acute kidney injury, *AFL* Atrial flutter/fibrillation, *AHT* Arterial hypertension, *LVHF* Left ventricular heart failure, *CKD* chronic kidney disease, *cL/RVHF* chronic left/right heart failure, *CeVD* cerebrovascular disease, *CABG* coronary artery bypass grafting, *CHD* coronary heart disease, *DM* diabetes mellitus, *DES* drug-eluting stent, *DEB* drug-eluting balloon, *LCH* left-heart catheterization, *LV* left ventricular, *MI* myocardial infarction, *PAD* peripheral artery disease, *POBA* plain old balloon angioplasty, *MI* previous myocardial infarction, *PCI* previous percutaneous coronary intervention

	♂ (1,315,700)	♀ (1,300,762)	total (2,616,462)
Cardiovascular risk factors			
DM	39.59% (*N* = 520,926)	36.78% (*N* = 478,361)	38.19%
dyslipidemia	34.22% (*N* = 450,298)	26.08% (*N* = 339,291)	30.18%
obesity	10.85% (*N* = 142,705)	11.21% (*N* = 145,844)	11.03%
smoking	2.89% (*N* = 38,008)	1.14% (*N* = 14,810)	2.02%
AHT	74.04% (*N* = 974,138)	78.21% (*N* = 1,017,280)	76.11%
cardiovascular comorbidities			
ischemic stroke	0.49% (*N* = 6,407)	0.49% (*N* = 6,350)	0.49%
PAD	7.38% (*N* = 97,111)	4.04% (*N* = 52,524)	5.72%
hemorrhagic stroke	0.12% (*N* = 1,576)	0.07% (*N* = 921)	0.10%
cRVHF	35.07% (*N* = 461,436)	34.88% (*N* = 453,667)	34.97%
cLVHF	1.57% (*N* = 20,681)	1.27% (*N* = 16,567)	1.42%
CKD	48.10% (*N* = 632,850)	49.10% (*N* = 638,687)	48.60%
CHD	42.68% (*N* = 561,517)	22.61% (*N* = 294,134)	32.70%
previous stroke	2.78% (*N* = 36,545)	2.91% (*N* = 37,852)	2.84%
AFl	53.67% (*N* = 706,082)	56.08% (*N* = 729,410)	54.86%
previous CABG	11.66% (*N* = 153,386)	3.99% (*N* = 51,933)	7.85%
CeVD	1.62% (*N* = 21,332)	1.09% (*N* = 14,235)	1.36%
previous valve	2.19% (*N* = 28,775)	1.68% (*N* = 21,902)	1.94%
previous MI	11.28% (*N* = 148,476)	6.51% (*N* = 84,733)	8.91%
previous PCI	22.01% (*N* = 289,641)	13.61% (*N* = 177,093)	17.84%
Recorded interventions			
LCH	21.65% (*N* = 284,852)	13.92% (*N* = 181,039)	17.81%
PCI	5.43% (*N* = 71,484)	2.60% (*N* = 33,814)	4.02%
DES	4.59% (*N* = 60,435)	2.19% (*N* = 28,436)	3.40%
DEB	0.37% (*N* = 4,918)	0.15% (*N* = 1,903)	0.26%
POBA	4.76% (*N* = 62,576)	2.20% (*N* = 28,558)	3.48%
CABG	0.08% (*N* = 1,053)	0.02% (*N* = 305)	0.05%
Complications			
cardiogenic shock	1.47% (*N* = 19,349)	0.87% (*N* = 11,300)	1.17%
AKI	11.37% (*N* = 149,657)	9.60% (*N* = 124,882)	10.49%
renal replacement therapy	2.93% (*N* = 38,546)	1.56% (*N* = 20,293)	2.25%
resuscitation + LV support	1.77% (*N* = 23,345)	1.03% (*N* = 13,437)	1.41%
assist device	0.19% (*N* = 2,556)	0.06% (*N* = 771)	0.13%
mechanical ventilation	7.01% (*N* = 92,262)	5.84% (*N* = 75,928)	6.43%
acute MI	2.8% (*N* = 36,821)	2.4% (*N* = 30,599)	2.6%

**Table 2:** In-hospital treatment and outcomes. In-hospital treatment and outcomes relative to the total number of male and female cases in hospital admissions for LVHF, presented in absolute and relative numbers, stratified by sex. Heart transplantation (HTx), transplantation (Tx)

	♂ (1,315,700)	♀ (1,300,762)	Total (2,616,462)
univentricular intracoporal pump	0.16% (*N* = 2,096)	0.03% (*N* = 357)	0.09%
biventricular intracoporal pump	≤ 0.01% (≤ 2)	≤ 0.01% (≤ 2)	≤ 0.01%
total artificial heart	< 0.01% (15)	< 0.01% (5)	< 0.01%
orthotropic HTx	≤ 0.02% (288)	0.01% (99)	0.01%
heterotropic HTx	0.00% (0)	0.00% (0)	< 0.01%
heart–lung-Tx	< 0.01% (≤ 8)	< 0.01% (≤ 8)	0.00%
death	7.9% (103,883)	8.34% (108,461)	8.12%

Male cases were more likely to have undergone prior cardiovascular interventions, such as valve implantation or PCI, and were more frequently subjected to invasive procedures during hospitalization, including L/RHC, stent implantation, POBA, and CABG (Table 1). Likewise, complications and adverse in-hospital outcomes occurred more frequently in male cases than in female cases. When stratified by NYHA class, the sex-specific differences in CVRF and cardiovascular comorbidities observed in the overall cohort were largely preserved within the individual NYHA groups as well (Online Resource 4). Exceptions included, for example, a higher proportion of female cases with DM in NYHA IV (♂: 41.95% vs. ♀: 48.50%), a greater prevalence of overweight male cases in NYHA I (♂: 9.95% vs. ♀: 8.81%), and fewer female cases with AFl in NYHA stages I and II. Female in-hospital mortality remained higher than that of male cases across all NYHA classes (Online Resource 3, Fig. 1).

**Fig. 1:**
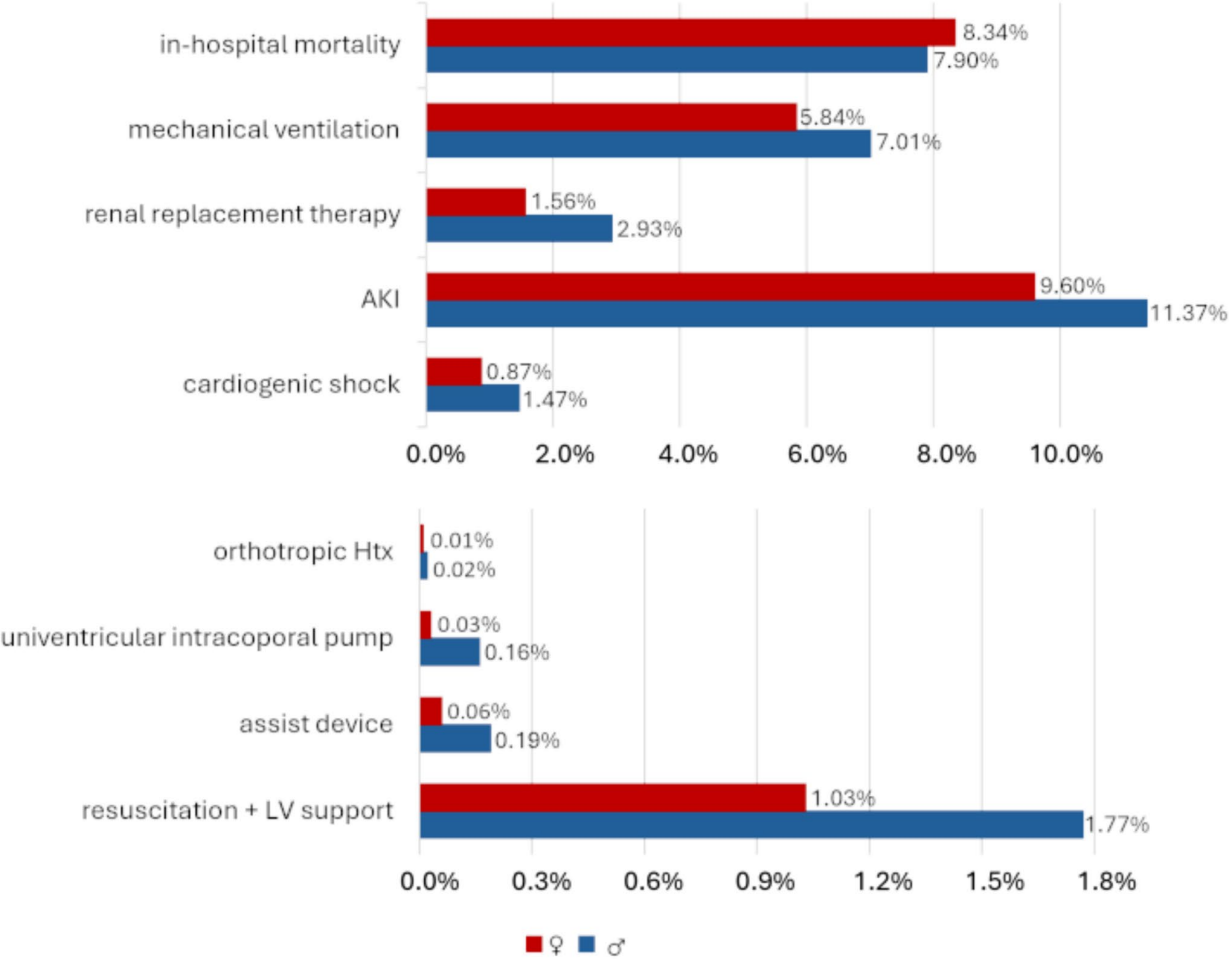
Occurrence of selected complications and outcomes by sex in cases with LVHF. Relative frequencies of in-hospital treatments and complications including cardiogenic shock, mechanical ventilation, AKI, renal replacement therapy, resuscitation with LV support and assist device, as well as outcomes including univentricular intracorporeal pump, HTx, and in-hospital mortality. Acute kidney injury (AKI), left ventricular heart failure (LVHF), heart transplantation (HTx), left ventricular (LV), New York Heart Association (NYHA)

**Fig. 2:**
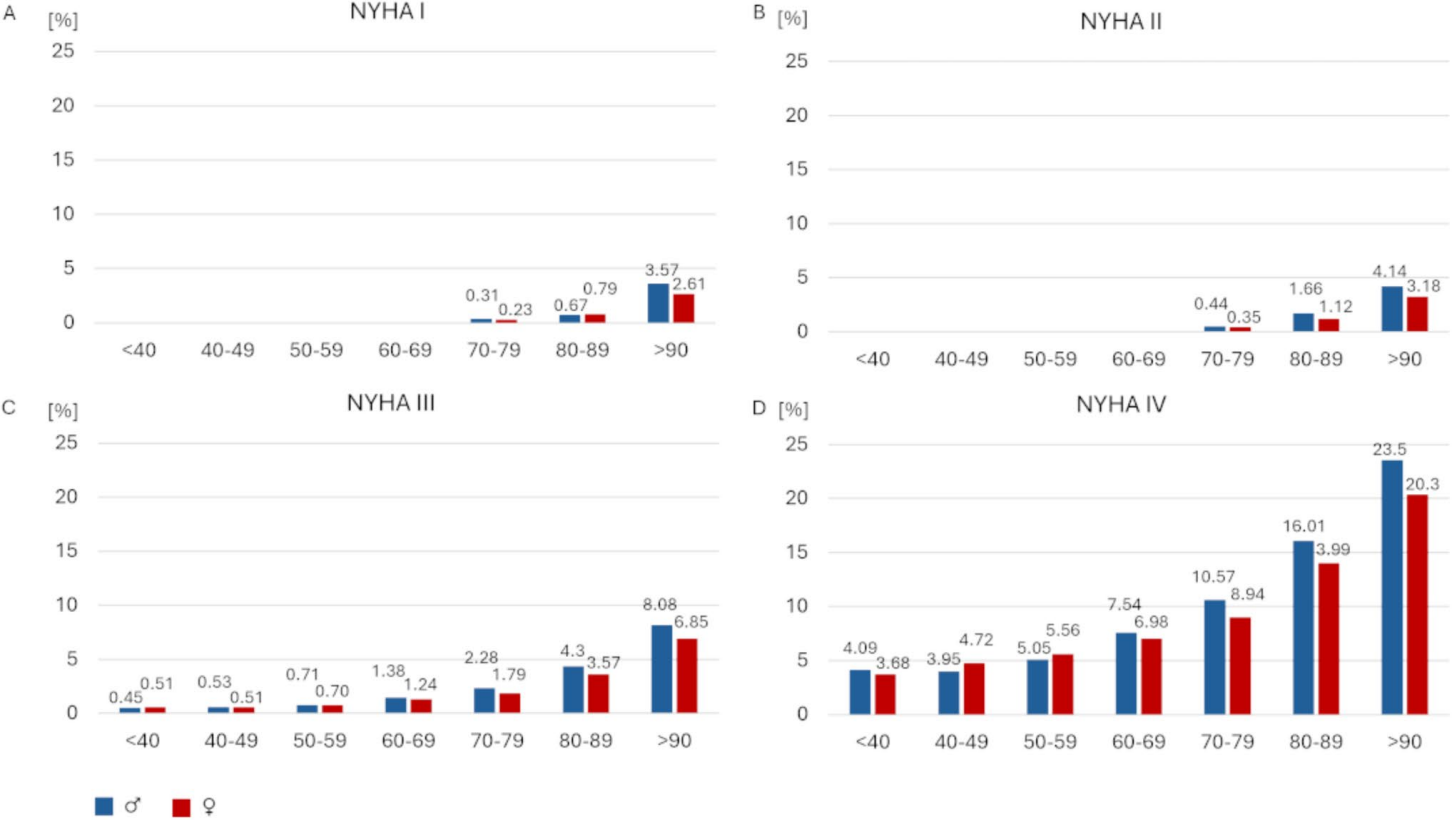
In-hospital mortality in cases with LVHF. In-hospital mortality in cases with LVHF, stratified by age, sex and NYHA classification (A: NYHA I, B: NYHA II, C: NYHA III; D: NYHA IV). Relative frequencies. New York Heart Association (NYHA), left ventricular heart failure (LVHF)

When stratifying not only by sex and NYHA stage but also by age group, we observed that the proportion of male cases who succumbed to in-hospital mortality exceeded that of female cases in most age and NYHA categories. However, exceptions were noted in specific subgroups: female cases aged 80–89 years in NYHA class I, those younger than 40 years in NYHA class III, as well as those aged 40–49 and 50–59 years in NYHA class IV exhibited higher mortality rates compared to their male counterparts (Fig. 2, Online Resource 5).

### Trends in median healthcare costs and hospitalization duration

The median duration of hospitalization, median age, and median healthcare costs increased with NYHA stage in both male and female cases (Online Resource 4). Across all NYHA classes, female cases were, on average, older than their male counterparts (Online Resource 4). While the median length of hospital stay was in median one day longer for female cases in NYHA stages I (♀: 4 vs. ♂: 3 days) and II (♀: 5 vs. ♂: 4 days), it was comparable between sexes in NYHA stages III (♀ = ♂: 7 days) and IV (♀ = ♂: 8 days) (Online Resource 4).

Our cohort data indicate that the treatment costs were consistently higher for male cases across in advanced LVHF (NYHA III: 2,931.34 vs. 2,922.67; NYHA IV: 2,960.72 vs. 2,923.36) (Online Resource 4).

### Multivariable logistic analysis of in-hospital mortality

**Fig. 3:**
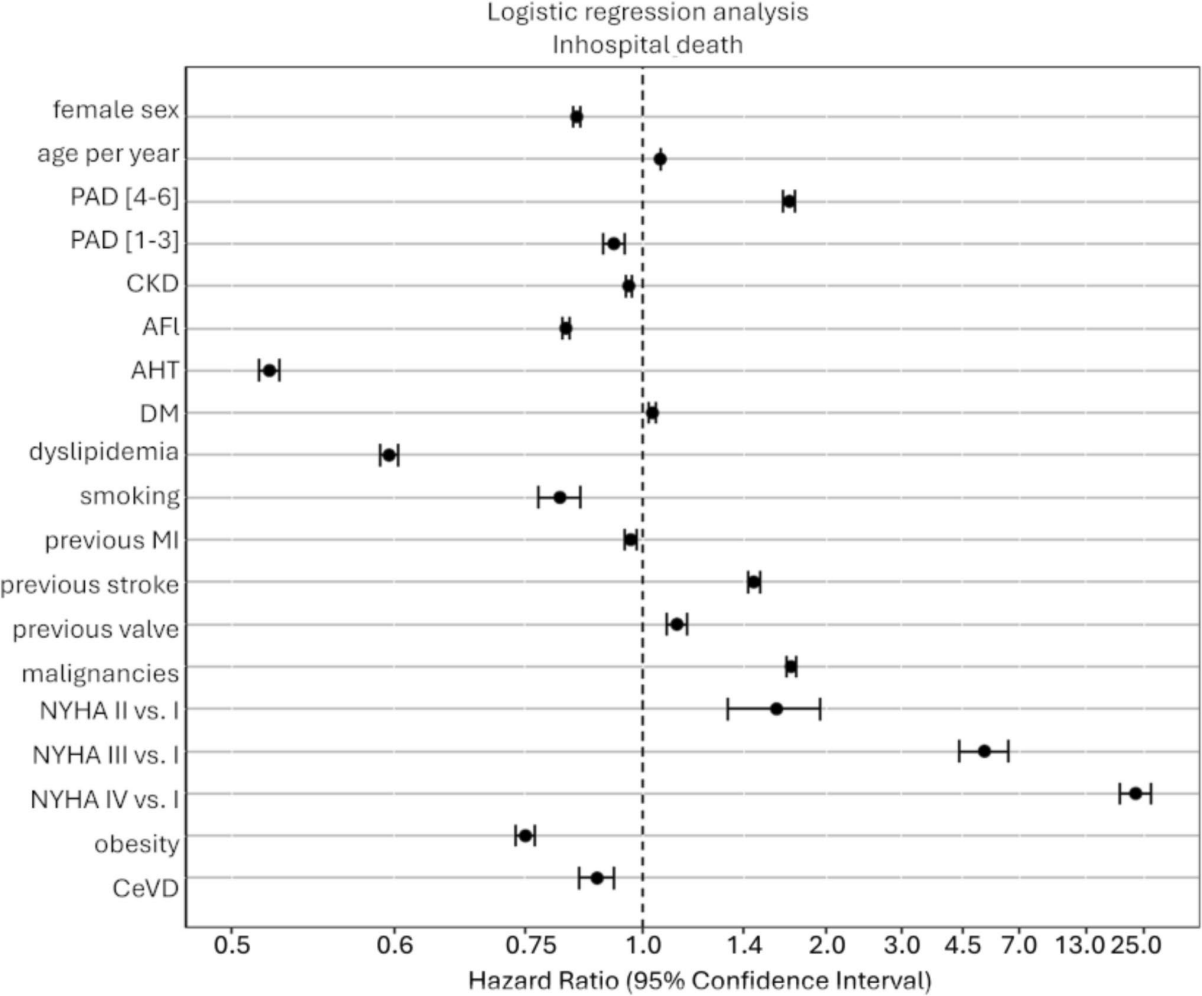
Multivariable logistic regression analysis for predicting in-hospital mortality. In-hospital mortality in cases with LVHF. The covariates included in the regression model were female sex, age, PAD (differentiated by the number of affected vessels), CKD, AFl, AHT, DM, dyslipidemia, smoking, previous MI, previous stroke, previous valve intervention, malignancies, NYHA classification, obesity, and CeVD. Atrial flutter/fibrillation (AFl), arterial hypertension (AHT), left ventricular heart failure (LVHF), chronic kidney disease (CKD), cerebrovascular disease (CeVD), diabetes mellitus (DM), peripheral artery disease (PAD), previous myocardial infarction (MI), New York Heart Association (NYHA)

We also applied a multivariable logistic analysis for in-hospital mortality including age, sex and baseline comorbidities and risk factors (i.e., PAD, CKD, AFl, AHT, DM, dyslipidemia, smoking, previous MI, previous stroke, previous valve, malignancies, NYHA stage, obesity and CeVD) (Fig. 3).

The analysis revealed that obesity (OR 0.751, [0.737;0.0.765]; *p* < 0.001) and female sex (OR 0.841, [0.833;0.849]; *p* < 0.001) were associated with a lower risk for in-hospital mortality, whereas the NYHA stage was a predictor for worse outcome (NYHA II: OR 1.596, [1.315;1.937]; *p* < 0.001; NYHA III: OR 5.290, [4.390;6,374]; *p* < 0.001; NYHA IV: OR 22.533, [18.704;27.145]; *p* < 0.001) (Fig. 3, Online Resource 6). The multivariable logistic regression model for in-hospital mortality showed good discrimination, with a c-statistic of 0.765 and Somers’ D of 0.53. The Hosmer-Lemeshow goodness-of-fit test yielded χ^2^ = 207.3, df = 8, *p* < 0.0001, which likely reflects the large sample size rather than poor model calibration.

## Discussion

Our study provides a comprehensive analysis of sex-specific differences in LVHF outcomes, emphasizing differences across NYHA strata. Female cases were more frequently classified in early NYHA stages, whereas female cases exhibited markedly higher in-hospital mortality rates in most age and NYHA-stratified groups.

The balanced sex distribution in our cohort contrasts with prior studies suggesting a male predominance in LVHF populations [11]. However, we observed that women were overrepresented in earlier NYHA stages, whereas men were more frequently classified as NYHA III. This aligns with previous reports that women are more likely to present with HFpEF, while men more commonly develop HFrEF [12], differences that may be associated with variations in disease severity and prognosis. Unfortunately, the nature of the available data (as there is no ICD coding to distinguish between HFpEF and HFrEF) does not allow for a differentiation between these two conditions; hence, further studies are warranted.

The pattern of NYHA classes observed in this nationwide inpatient dataset contrasts with distributions reported in prospective HF cohorts and clinical registries, where the majority of hospitalized patients are commonly assigned to NYHA class III, and NYHA class IV accounts for a smaller fraction of cases [13–16]. This discrepancy is likely related to differences in how NYHA class is assessed and recorded across data sources. In routine hospital documentation, NYHA class is often recorded in the context of the acute admission and may therefore reflect transient functional limitation during decompensation rather than the patient’s habitual functional capacity in the stable outpatient setting. Consequently, NYHA in the present analysis should be viewed primarily as a marker of clinical severity at hospital presentation rather than as a surrogate of long-term disease stage. This interpretation is particularly pertinent given that NYHA showed the strongest association with in-hospital mortality in the regression models, indicating that the models predominantly account for short-term severity of illness at admission rather than baseline HF severity.

CVRF were highly prevalent in the cohort, with AHT, DM, and dyslipidemia being the most common comorbidities. Consistent with existing literature, male cases had a higher burden of cardiovascular comorbidities and were more likely to undergo invasive procedures, such as PCI and CABG. In contrast, female cases exhibited higher rates of CKD, obesity, AHT, prior stroke, and AFl - conditions known to influence LVHF prognosis. These comorbidities are particularly relevant in the context of HFpEF, which is more prevalent in women and has distinct pathophysiological mechanisms compared to HFrEF.

The interpretation of sex-specific differences in mortality requires careful consideration of both unadjusted and adjusted results. In our unstratified population-level analysis, female cases showed a higher overall in-hospital mortality than male cases, which is consistent with prior literature [3, 4, 14, 17] and likely reflects differences in age distribution and comorbidity profiles. However, in the multivariable logistic regression model - which adjusts for age, NYHA stage and major comorbidities - female sex was associated with a lower risk of in-hospital mortality. These findings should be interpreted as descriptive associations rather than evidence of causal effects. When further stratified by NYHA class and age, male cases demonstrated higher mortality rates in most subgroups, while female cases exhibited higher mortality only in specific segments (e.g., younger female cases in NYHA III–IV and female cases aged 80–89 with NYHA I). These patterns underscore the heterogeneity of LVHF and suggest that observed sex-related differences in mortality may vary according to age, functional status, and comorbidity burden.

Several factors may be associated with these divergent trajectories. Differences in frailty, healthcare access and treatment responses among elderly and younger female patients may play an important role. Moreover, well-described sex-based differences in pharmacokinetics and pharmacodynamics - such as variations in drug absorption, distribution, metabolism and receptor sensitivity - may further influence clinical outcomes [18–20]. Women remain consistently underrepresented in cardiovascular trials, resulting in guideline recommendations that are largely based on male-centric evidence [21–23]. This imbalance is reflected in real-world care, where women receive guideline-directed therapies, including RAAS inhibitors and beta-blockers, less frequently than men [3, 4, 17]. Such disparities in care may be associated with differences in observed outcomes across specific female subgroups.

Given that our dataset does not include key clinical variables such as LVEF or detailed pharmacological treatment data, the present results must be interpreted as descriptive associations rather than causal conclusions. Nevertheless, they highlight clinically vulnerable subgroups and underscore the need for more granular, phenotype-specific studies integrating echocardiographic data, treatment patterns, biological determinants and sex-associated pharmacological effects to better understand and address these disparities in LVHF outcomes.

Economic analyses revealed that male cases incurred higher CM adjusted healthcare costs across in advanced NYHA stages, despite being hospitalized for a median duration equal to or shorter than female cases and being younger than female cases on average. This may be related to their greater utilization of advanced therapies and interventions, as well as higher rates of hospital complications.

Finally, our multivariable logistic analysis confirmed that NYHA stage remains the strongest predictor of in-hospital mortality, with a stepwise increase in risk from NYHA II to IV. Female sex and obesity were associated with lower odds of in-hospital mortality, consistent with prior studies suggesting an "obesity paradox" in LVHF. These associations should not be interpreted causally but may inform hypothesis generation regarding potential modifiers of risk across sex and disease severity strata.

A major strength of the present study lies in the comprehensive nationwide coverage of hospitalized LVHF cases in a large, industrialized country, allowing an unselected and real-world description of sex- and NYHA-specific outcome patterns. Such population-level data are essential for hypothesis generation and for identifying clinically relevant disparities that warrant further investigation in more granular, mechanistic studies.

Our study underscores the need for personalized LVHF management that accounts for sex and disease severity. However, these associations must be interpreted cautiously in light of the lack of key prognostic variables, including LVEF and detailed treatment information. Consequently, our results cannot exclude the possibility that the observed differences are at least partly explained by these unmeasured factors. The observed differences may, at least in part, reflect unmeasured phenotypic and therapeutic factors. The higher prevalence of selected comorbidities among women highlights the importance of comprehensive, sex-sensitive risk factor management. Future research should aim to integrate detailed clinical, echocardiographic, and treatment data to validate and refine these observations and to develop targeted strategies that ensure equitable care for all LVHF patients.

## Limitations

When interpreting the presented data, several limitations must be taken into account. First, important clinical parameters such as LVEF are not available in the DESTATIS dataset, as echocardiographic findings are not part of the administrative coding. Given the well-established prognostic role of LVEF in HF, this absence may limit the ability to fully adjust for disease severity and hampers stratification by HF phenotype (HFrEF, HFmrEF, HFpEF). Second, information on drug treatment - both outpatient prescriptions prior to admission and in-hospital administration - is likewise not included in the dataset. As guideline-directed medical therapy is a key determinant of outcome in HF, the lack of medication data restricts our ability to account for its potential impact on prognosis**.** Third, laboratory values at admission, including natriuretic peptides (NT-proBNP), hemoglobin, creatinine, and electrolytes, are not recorded in the dataset, which limits the clinical characterization of the study population. The findings are therefore to be interpreted in the context of these missing variables. As a result, the present analyses are descriptive in nature and do not permit causal inference; residual confounding by unmeasured clinical and treatment-related factors is likely. Further, the findings are specific to Germany, making them most applicable within this national framework and potentially relevant only to countries with comparable healthcare systems. The analysis is limited to in-hospital data, without insights into outpatient care or post-discharge outcomes, restricting the ability to fully assess LVHF management and patient recovery. Importantly, NYHA classification in administrative inpatient data is influenced by routine documentation and coding practices and may predominantly reflect functional impairment at the time of hospital admission rather than stable baseline functional class. Additionally, hospitalization data are case-based rather than patient-based, meaning that all hospitalizations within a year are counted without distinguishing unique individuals. While this does not affect the accuracy of the absolute number of in-hospital deaths, it prevents precise conclusions regarding the number of distinct patients affected. Other limitations include potential coding inaccuracies and variations in coding practices over time, particularly in relation to LVHF with preserved ejection fraction. Moreover, external factors such as evolving treatment guidelines and public health measures may have influenced the observed trends. Finally, data on crucial outcomes such as quality of life and readmission rates are not available, and unmeasured factors, including socioeconomic status, could introduce bias. These considerations should be kept in mind when interpreting the study’s findings and assessing their broader applicability.

## Conclusion

Our study underscores the need for a personalized approach to LVHF management that considers both sex and disease severity. The observed sex-related differences in in-hospital mortality highlight the relevance of recognising clinically vulnerable subgroups. While multivariable logistic analyses showed an association between female sex and lower odds of in-hospital mortality, age- and NYHA-stratified analyses identified specific subgroups in which female cases exhibited higher mortality, challenging the notion of a uniform survival advantage among women. However, as information on LVEF and pharmacological treatment was not available, these results should be interpreted as associative and potentially influenced by unmeasured confounding. Further studies incorporating detailed echocardiographic and medication data are warranted to confirm and extend these findings. Moreover, the higher prevalence of selected comorbidities among women emphasizes the importance of comprehensive, sex-sensitive risk factor management. Future research should focus on optimizing treatment strategies, tailoring medical therapies, and addressing the underrepresentation of women in clinical trials to ensure equitable care for all LVHF patients.

## Supplementary Information

Below is the link to the electronic supplementary material.

ESM 1 (DOCX 57.4 KB)

## Data Availability

The datasets analyzed in the current study are not publicly available due to legal and ethical restrictions imposed by German data protection law. The data were accessed via DESTATIS. Qualified researchers may apply for data access through DESTATIS, subject to formal approval procedures and data protection requirements. The authors do not have permission to share the underlying individual-level data.

## Declarations

The investigation conforms with the principles outlined in the *Declaration of Helsinki* (Br Med J 1964; ii:177). Patient consent and ethical approval were waived due to the retrospective character with anonymized data of this study and all the procedures being performed were part of the routine care.
