# Supplementary material for: Sex-specific differences in disease severity and outcomes in left ventricular heart failure: a nationwide cohort study
Source: Clin Res Cardiol. 2026 Feb 23;115(9):1653–63. doi: 10.1007/s00392-026-02876-9 (PMC13457246; doi:10.1007/s00392-026-02876-9)
Supplement: Supplementary file 1 [file 392_2026_2876_MOESM1_ESM.docx]

# **Sex-Specific Differences in Disease Severity and Outcomes in Left Ventricular Heart Failure: A Nationwide Cohort Study**

Anastasia Janina Hobbach, MD^1^, Jannik Feld, PhD^2^, Jeanette Köppe, PhD^2^, Jürgen Reinhard Sindermann, MD^1^, Holger Reinecke, MD^1^

^1^Department of Cardiology I, Coronary, Peripheral Vascular Disease and Heart Failure, University Hospital Münster, Münster, Germany

^2^Institute of Biostatistics and Clinical Research, University of Münster, Münster, Germany

Correspondence to Anastasia J. Hobbach

^1^Department of Cardiology I, Coronary, Peripheral Vascular Disease and Heart Failure, University Hospital Münster, Albert-Schweizer Campus 1, 48149 Münster, Germany

ORCID: 0000-0001-6421-8429

Tel: +49 251 83 44234

Email: Anastasia.Hobbach@ukmuenster.de

**Online Resource 1:** ICD-10-GM Codes used to define health conditions.

| Health Problems 10th Revision (ICD-10 GM) | Code |
| --- | --- |
| Arterial hypertension (AHT) | I10.-, I11.-, I12.-, I13.-, I15.- |
| Diabetes mellitus (DM) | E10.-, E11.-, |
| Dyslipidemia | E78.- |
| smoking | F17.- |
| Previous Myocardial Infarction (MI) | I25.2, I24.1 |
| Acute Myocardial Infarction (MI) | I21.-, I22.- |
| Obesity | E66.- |
| Chronic right heart failure (cRVHF) | I50.0- |
| Chronic left heart failure (cLVHF) | I50.11-I50.14 |
| Primary RVHF | I50.0, I50.1 |
| Global or secondary RVHF due to LVHF | I50.01 |
| Unspecified heart failure | I50.9 |
| Previous stroke | I69.3, I69.4 |
| Coronary heart disease (CHD) | I25.- |
| Atrial flutter/fibrillation (AFl) | I48.- |
| Peripheral artery disease (PAD) | I70.2- |
| Cerebrovascular disease (CeVD) | I65.-, |
| Chronic kidney disease (CKD) | N18.-, N19.- |
| Acute kidney injury (AKI) | N17 |
| Cardiogenic shock | R57.0, T81.1 |

**Online Resource 2:** OPS codes used to identify medical procedures (German Procedure Classification System).

| German procedure classification system (OPS) | Code |
| --- | --- |
| Percutaneous coronary intervention (PCI) | 8-837 |
| Left-heart-Catheterization (LCH) | 1-275 |
| Biventricular intracoporal pump | 5-376.50 \| |
| Univentricular intracoporal pump | 5-376.40 |
| Impella | 8-83a.3 |
| Artificial heart | 5-376.6 \| |
| HTx heterotopic | 5-375.1 |
| HTx orthotopic | 5-375.0 |
| Heart- and Lung Tx | 5-375.2 |
| Intra-aortic ballon pump (IABP) | 8-83a.0 |
| Renal replacement therapy | 8-853, 8-855, 8-854, 8-857 |
| Left ventricle support | 8-851, 8-852, 8-83a.0, 8-83a.3 |
| Resuscitation | 8-771 |

**Online Resource 3: Cardiovascular risk factors, cardiovascular comorbidities, recorded intervention, complications and outcome stratified by sex and NYHA classifications.** Distribution of cases with LVHF according to NYHA stages; as well as distribution of cardiovascular risk factors, cardiovascular comorbidities, recorded intervention, complications and outcome stratified by sex. Absolute and relative frequencies**.** Acute kidney injury (AKI), atrial flutter/fibrillation (AFL), arterial hypertension (AHT), bare-metal stent (BMS), left ventricular heart failure (LVHF), chronic kidney disease (CKD), chronic right ventricular heart failure (CRHF), cerebrovascular disease (CeVD), coronary artery bypass grafting (CABG), coronary heart disease (CHD), diabetes mellitus (DM), drug-eluting stent (DES), drug-eluting balloon (DEB), heart transplantation (HTx), left-heart catheterization (LCH), left ventricular (LV), myocardial infarction (MI), peripheral artery disease (PAD), plain old balloon angioplasty (POBA), previous myocardial infarction (MI), previous percutaneous coronary intervention (PCI), transplantation (Tx).

|  | NYHA I | | NYHA II | | NYHA III | | NYHA IV | |
| --- | --- | --- | --- | --- | --- | --- | --- | --- |
| % (N) | 0.98% (25,698) | | 6.42% (167,918) | | 40.39% (1,056,912) | | 52.21% (1,365,934) | |
|  | ♂ | ♀ | ♂ | ♀ | ♂ | ♀ | ♂ | ♀ |
| % (N) | 44.05%  (11,321) | 55.95%  (14,377) | 48.84%  (82,010) | 51.16%  (85,908) | 51.14%  (540,518) | 48.86%  (516,394) | 49.92%  (681,851) | 50.08%  (684,083) |
| Cardiovascular risk factors |  |  |  |  |  |  |  |  |
| DM % (N) | 24.15%  (2,734) | 22.57%  (3,245) | 30.06%  (24,649) | 27.59%  (23,703) | 38.39%  (207,495) | 35.25%  (182,047) | 41.95%  (286,048) | 48.50%  (269,366) |
| dyslipidemia % (N) | 36.53%  (4,136) | 30.59%  (4,398) | 39.39%  (32,302) | 31.70%  (27,236) | 36.59%  (197,752) | 28.00%  (144,608) | 31.69%  (216,108) | 23.83%  (163,049) |
| obesity % (N) | 9.95% (1,127) | 8.81% (1,266) | 11.59% (9,508) | 11.69% (10,041) | 11.51% (62,237) | 11.83% (61,076) | 10.24% (69,833) | 10.74% (73,461) |
| smoking % (N) | 3.03%  (343) | 1.41%  (203) | 3.6%  (2,954) | 1.6%  (1,375) | 2.79% (15,061) | 1.13%  (5,838) | 2.88% (19,650) | 1.08%  (7,394) |
| AHT % (N) | 85.23% (9,649) | 92.45% (13,291) | 78.41% (64,306) | 83.84% (72,029) | 75.26% (406,783) | 79.28% (409,420) | 72.36% (493,400) | 76.39% (522,540) |
| cardiovascular comorbidities |  |  |  |  |  |  |  |  |
| ischemic stroke % (N) | 0.24%  (27) | 0.2%  (29) | 0.21%  (171) | 0.21%  (178) | 0.36%  (1,920) | 0.36%  (1,857) | 0.63%  4,289) | 0.63%  4,286) |
| PAD % (N) | 3.71%  (420) | 2.03%  (292) | 4.64% (3,804) | 2.52% (2,167) | 6.72% (36,337) | 3.68% (18,986) | 8.29% (56,550) | 4.54% (31,079) |
| hemorrhagic stroke % (N) | 0.03%  (3) | 0.04%  (6) | 0.06%  (51) | 0.03%  (24) | 0.08%  (443) | 0.05%  (265) | 0.16%  (1,079) | 0.09%  (626) |
| CRHF % (N) | 6.03%  (683) | 5.49%  (790) | 13.27% (10,879) | 13.43% (11,534) | 31.52% (170,395) | 31.91% (164,761) | 40.99% (279,479) | 40.43% (276,582) |
| LVHF % (N) | 1.03%  (117) | 0.75%  (108) | 1.45% (1,189) | 1.13%  (968) | 1.47%  (7,950) | 1.2%  (6,187) | 1.68% (11,425) | 1.36%  (9,304) |
| CKD % (N) | 20.86% (2,362) | 22.41% (3,222) | 29.73% (24,381) | 33.03% (28,377) | 46.05% (248,908) | 47.90% (247,361) | 52.39% (357,199) | 52.59% (359,727) |
| CHD % (N) | 28.71% (3,250) | 14.13% (2,032) | 39.93% (32,748) | 20.21% (17,358) | 44.11% (238,403) | 23.44% (121,056) | 42.11% (287,116) | 22.47% (153,688) |
| previous stroke % (N) | 1.55%  (175) | 1.35%  (194) | 1.59% (1,302) | 1.63% (1,403) | 2.23% (12,075) | 2.4%  (12,399) | 3.37% (22,993) | 3.49% (23,856) |
| AFl % (N) | 23.48% (2,658) | 21.17% (3,043) | 38.06% (31,214) | 37.93% (32,581) | 54.27% (293,362) | 57.36% (296,204) | 55.56% (378,848) | 58.12% (397,582) |
| previous CABG % (N) | 5.21%  (590) | 1.71%  (246) | 8.42% (6,907) | 2.79% (2,400) | 12.08% (65,291) | 4.08% (21,066) | 11.82% (80,598) | 4.13% (28,221) |
| CeVD % (N) | 1.64%  (186) | 1.54%  (222) | 1.62% (1,325) | 1.16%  (999) | 1.55%  (8,362) | 1.06%  (5,482) | 1.68% (11,459) | 1.1%  (7,532) |
| previous valve % (N) | 1.14%  (129) | 0.68%  (98) | 1.81% (1,484) | 1.23% (1,055) | 2.24% (12,104) | 1.71%  (8,811) | 2.21% (15,058) | 1.75% (11,938) |
| previos MI % (N) | 6.83%  (773) | 3.61%  (519) | 9.46% (7,759) | 5.03% (4,321) | 11.13% (60,160) | 6.2%  (32,013) | 11.7% (79,784) | 7% (47,880) |
| previous PCI % (N) | 7.1%  (804) | 5.14%  (739) | 13.76% (11,287) | 9.36% (8,043) | 22.21% (120,044) | 14.16% (73,133) | 23.1% (157,506) | 13.91% (95,178) |
| Recorded interventions |  |  |  |  |  |  |  |  |
| LCH % (N) | 28.72% (3,251) | 17.47% (2,512) | 31.21% (29,697) | 26.61% (22,857) | 25.05% (135,426) | 16.71% (86,284) | 17.08% (116,478) | 10.14% (69,386) |
| PCI % (N) | 3.94%  (446) | 1.22%  (175) | 7.25% (5,948) | 2.77% (2,376) | 6.18% (33,421) | 3.01% (15,544) | 4.64% (31,669) | 2.3%  (15,719) |
| DES % (N) | 3,41%  (386) | 1.09%  (156) | 6.31% (5,176) | 2.37% (2,040) | 5.25% (28,399) | 2.55% (13,143) | 3.88% (26,474) | 1.91% (13,097) |
| BMS % (N) | 0.16%  (18) | 0.02%  (3) | 0.25%  (204) | 0.11%  (97) | 0.28%  (1,507) | 0.15%  (773) | 0.24%  (1,614) | 0.11%  (774) |
| DEB % (N) | 0.15%% (17) | 0.06%  (8) | 0.52%  (424) | 0.16%  (136) | 0.45%  (2,431) | 0.18%  (938) | 0.3%  (2,046) | 0.12%  (821) |
| POBA % (N) | 3.59%  (406) | 1.03%  (148) | 6.48% (5,312) | 2.37% (2,034) | 5.39% (29,134) | 2.53% (13,084) | 4.07% (27,724) | 1.94% (13,292) |
| CABG % (N) | 0.19%  (22) | 0.02%  (3) | 0.12%  (95) | 0.03%  (23) | 0.08%  (408) | 0.02%  (106) | 0.08%  (528) | 0.03%  (173) |
| Complications |  |  |  |  |  |  |  |  |
| cardiogenic shock % (N) | 0.03%  (3) | 0.06%  (8) | 0.06%  (50) | 0.06%  (55) | 0.39%  (2,095) | 0.24%  (1,238) | 2.52% (17,201) | 1.46%  (9,999) |
| AKI % (N) | 1.73%  (196) | 1.61%  (231) | 3.14% (2,575) | 2.89% (2,486) | 7.48% (40,439) | 6.58% (33,963) | 15.61% (106,447) | 12.89% (88,202) |
| renal replacement therapy % (N) | 1.61%  (182) | 0.69%  (99) | 0.86%  (703) | 0.38%  (329) | 1.54%  (8,333) | 0.82%  (4,209) | 4.3%  (29,328) | 2.29% (15,656) |
| resuscitation + LV support % (N) | 0.1%  (11) | 0.08%  (11) | 0.21%  (176) | 0.12%  (104) | 0.74%  (4,026) | 0.44%  (2,281) | 2.81% (19,132) | 1.61% (11,041) |
| assist device % (N) | 0.00%  (0) | 0.00%  (0) | 0.03%  (27) | 0.01%  (6) | 0.08%  (427) | 0.03%  (137) | 0.31%  (2,102) | 0.09%  (628) |
| mechanical ventilation % (N) | 0.19%  (22) | 0.24%  (34) | 0.49%  (398) | 0.37%  (314) | 1.82%  (9,815) | 1.49%  (7,671) | 12.03% (82,027) | 9.93% (67,909) |
| Outcomes |  |  |  |  |  |  |  |  |
| univentricular intra-coporal pump % (N) | 0.00%  (0) | 0.00%  (0) | <0.01%  (4) | 0.00%  (0) | 0.06%  (307) | 0.01%  (36) | 0.26%  (1,785) | 0.05%  (321) |
| biventricular intra-coporal pump % (N) | ≤0.01%  (≤2) | ≤0.01%  (≤2) | ≤0.01%  (≤2) | ≤0.01%  (≤2) | ≤0.01%  (≤2) | ≤0.01%  (≤2) | ≤0.01%  (≤2) | ≤0.01%  (≤2) |
| total artificial heart % (N) | ≤0.01%  (≤2) | ≤0.01%  (≤2) | ≤0.01%  (≤2) | ≤0.01%  (≤2) | ≤0.01%  (≤2) | ≤0.01%  (≤2) | <0.01%  (15) | <0.01%  (5) |
| orthotropic Htx % (N) | 0.00%  (0) | 0.00%  (0) | ≤0.01%  (≤2) | ≤0.01%  (≤2) | 0.01%  (67) | ≤0.01%  (23) | 0.03%  (221) | ≤0.01%  (76) |
| heterotropic HTx % (N) | 0.00%  (0) | 0.00%  (0) | 0.00%  (0) | 0.00%  (0) | 0.00%  (0) | 0.00%  (0) | 0.00%  (0) | 0.00%  (0) |
| heart-lung-Tx % (N) | ≤0.02%  (≤2) | ≤0.01%  (≤2) | <0.01%  (≤2) | <0.01%  (2) | <0.01%  (≤2) | <0.01%  (≤2) | <0.01%  (≤2) | <0.01%  (≤2) |
| in-hospital mortality % (N) | 0.33%  (37) | 0.51%  (74) | 0.77%  (632) | 0.84%  (720) | 3.03% (16,391) | 3.24% (16,755) | 12.73% (86,823) | 13.29% (90,912) |

**Online Resource 4: Median healthcare costs, length of hospitalization, and age in LVHF cases stratified by sex.** Median healthcare costs in euro, median length of hospitalization due to LVHF in days with interquartile range (IQR), and median age in years in brackets, stratified by sex. NYHA: New York heart Association.

|  | sex | Median costs  [euro] | length of hospitalization  [days] | age  [years] |
| --- | --- | --- | --- | --- |
| NYHA I | ♂ | 2497.91 [1667.01;2931.34] | 3 [2;6] | 68 [57;78] |
|  | ♀ | 2758.86 [1994.98;2931.34] | 4 [2;6] | 76 [66;83] |
| NYHA II | ♂ | 2758.86 [1969.57;2970.32] | 4 [2;7] | 74 [63;81] |
|  | ♀ | 2758.86 [2038.53;2963.45] | 5 [2;7] | 79 [70;84] |
| NYHAIII | ♂ | 2931.34 [2498.01;3582.98] | 7 [4;10] | 78 [69;83] |
|  | ♀ | 2922.67 [2497.91;3140.55] | 7 [5;11] | 82 [76;87] |
| NYHA IV | ♂ | 2960.72 [2505.84;4320.66] | 8 [5;14] | 79 [70;84] |
|  | ♀ | 2923.36 [2498.48;3397.92] | 8 [5;13] | 83 [77;88] |

**Online Resource 5: In-hospital mortality in cases with LVHF.** In-hospital mortality in cases with LVHF, stratified by age, sex and NYHA classification. Case numbers and relative frequencies of cases who died during hospitalization. New York Heart Association (NYHA), left ventricular heart failure (LVHF).

|  |  | NYHA I |  | NYHA II |  | NYHA III |  | NYHA IV |  |
| --- | --- | --- | --- | --- | --- | --- | --- | --- | --- |
| age [years] |  | **♂** | **♀** | **♂** | **♀** | **♂** | **♀** | **♂** | **♀** |
| <40 | %  (N) | ≤ 0,001% | ≤ 0,001% | ≤ 0,001% | ≤ 0,001% | 0.45%  (3,753) | 0.51%  (1780) | 4.09%  (4331) | 3.68%  (1683) |
| 40-49 | %  (N) | ≤ 0,001% | ≤ 0,001% | ≤ 0,001% | ≤ 0,001% | 0.53% (10,496) | 0.51%  (3,895) | 3.95%  (10,737) | 4.72%  (3,664) |
| 50-59 | %  (N) | ≤ 0,001% | ≤ 0,001% | ≤ 0,001% | ≤ 0,001% | 0.71% (39,938) | 0.7%  (15,092) | 5.05%  (41,923) | 5.56%  (14,689) |
| 60-69 | %  (N) | ≤ 0,001% | ≤ 0,001% | ≤ 0,001% | ≤ 0,001% | 1.38% (87,076) | 1.24% (43,393) | 7.54% (101,003) | 6.98%  (48,899) |
| 70-79 | %  (N) | 0.31% (2,940) | 0.23% (4,362) | 0.44% (25,805) | 0.35% (26,667) | 2.28% (174,334) | 1.79% (138,246) | 10.57% (209,843) | 8.94% (156,038) |
| 80-89 | %  (N) | 0.67% (2,097) | 0.76% (4,600) | 1.66% (21,091) | 1.12% (32,176) | 4.3% (193,361) | 3.57% (240,619) | 16.01% (260,061) | 13.99% (328,188) |
| >90 | %  (N) | 3.57%  (308) | 2.61%  (959) | 4.14%  (2,847) | 3.18%  (7,253) | 8.08% (31,560) | 6.85% (73,369) | 23.5%  (53,953) | 20.3% (130,922) |

|  | Odds-Ratio | 95% Wald  Confidence limits |
| --- | --- | --- |
| female sex | 0.841 | [0.833;0.849] |
| age | 1.052 | [1.052;1.053] |
| PAD 4-6 | 1.687 | [1.644;1.731] |
| PAD 1-3 | 0.923 | [0.898;0.949] |
| CKD | 0.961 | [0.952;0.970] |
| AFl | 0.820 | [0.813;0.828] |
| AHT | 0.518 | [0.513;0.523] |
| DM | 1.028 | [1.018;1.038] |
| dyslipidemia | 0.596 | [0.589;0.604] |
| smoking | 0.809 | [0.772;0.848] |
| previous MI | 0.966 | [0.949;0.983] |
| previous stroke | 1.454 | [1.421;1.489] |
| previous valve | 1.108 | [1.072;1.146] |
| malignancies | 1.699 | [1.663;1.735] |
| NYHA II vs. I | 1.596 | [1.315;1.937] |
| NYHA III vs. I | 5.290 | [4.39;6.374] |
| NYHA IV vs. I | 22.533 | [18.704;27.147] |
| obesity | 0.751 | [0.737;0.765] |
| CeVD | 0.884 | [0.847;0.923] |

**Online Resource 6: Odds-Ratio and confidence interval for the multivariable Cox regression analysis for predicting in-hospital mortality.** Atrial flutter/fibrillation (AFL), arterial hypertension (AHT), cerebrovascular disease (CeVD), chronic kidney disease (CKD), diabetes mellitus (DM), myocardial infarction (MI), peripheral artery disease (PAD), New York Heart Association (NYHA).
